# Predicting Kawasaki disease shock syndrome in children

**DOI:** 10.3389/fimmu.2024.1400046

**Published:** 2024-06-03

**Authors:** Zhihui Zhao, Yue Yuan, Lu Gao, Qirui Li, Ying Wang, Shunying Zhao

**Affiliations:** Beijing Children’s Hospital, Capital Medical University, National Center for Children’s Health, Beijing, China

**Keywords:** Kawasaki disease shock syndrome, Kawasaki disease, logistic regression model, nomogram model, early identification

## Abstract

**Background:**

Kawasaki disease shock syndrome (KDSS) is a critical manifestation of Kawasaki disease (KD). In recent years, a logistic regression prediction model has been widely used to predict the occurrence probability of various diseases. This study aimed to investigate the clinical characteristics of children with KD and develop and validate an individualized logistic regression model for predicting KDSS among children with KD.

**Methods:**

The clinical data of children diagnosed with KDSS and hospitalized between January 2021 and December 2023 were retrospectively analyzed. The best predictors were selected by logistic regression and lasso regression analyses. A logistic regression model was built of the training set (n = 162) to predict the occurrence of KDSS. The model prediction was further performed by logistic regression. A receiver operating characteristic curve was used to evaluate the performance of the logistic regression model. We built a nomogram model by visualizing the calibration curve using a 1000 bootstrap resampling program. The model was validated using an independent validation set (n = 68).

**Results:**

In the univariate analysis, among the 24 variables that differed significantly between the KDSS and KD groups, further logistic and Lasso regression analyses found that five variables were independently related to KDSS: rash, brain natriuretic peptide, serum Na, serum P, and aspartate aminotransferase. A logistic regression model was established of the training set (area under the receiver operating characteristic curve, 0.979; sensitivity=96.2%; specificity=97.2%). The calibration curve showed good consistency between the predicted values of the logistic regression model and the actual observed values in the training and validation sets.

**Conclusion:**

Here we established a feasible and highly accurate logistic regression model to predict the occurrence of KDSS, which will enable its early identification.

## 1 Introduction

Kawasaki disease (KD), also known as cutaneous mucosal lymph node syndrome, is an acute autoimmune vasculitis. KD, which usually occurs in children < 5 years of age, is an acute self-limited febrile disease characterized by a combination of several characteristic clinical symptoms, including polymorphic rash, nonsuppurative conjunctivitis, red mouth and lips, myrtle tongue, erythema and edema of the hands and feet, and cervical lymphedema (1). In addition to the characteristics of KD, patients with Kawasaki disease shock syndrome (KDSS) show poor perfusion or systolic hypotension. The systolic blood pressure of affected children is persistently lower (by ≥20%) than the normal low value of systolic blood pressure of children of the same age and requires volume expansion or vasoactive drugs to maintain blood pressure within the normal range.

The cause of KDSS hypotension is currently not fully understood. Many studies have shown that systemic vasculitis changes in acute KD can lead to persistent capillary leakage, abnormal cardiac systolic function, and abnormal regulation of inflammatory cytokines, among other issues. Many factors may contribute to KDSS hypotension (2). Maddox et al. calculated the data of KD patients from four large medical databases in the United States, 2006–2018, and found that the incidence of KDSS was 2.8–5.3%, and showing an upward trend in recent years (3). The early symptoms of KD may be atypical in some children with KDSS (4). Children with KDSS usually experience rapid progression and shock, often have stronger inflammatory reactions, and can be prone to coronary artery disease and multi-organ dysfunction, so its early recognition is particularly important. The pathogenesis of KDSS is complex and may be related to capillary leakage and decreased peripheral vascular resistance (5, 6), which cannot be predicted by a single index.

Therefore, this study aimed to build a KDSS risk prediction model for children with KD, identify the best risk prediction tool, improve the prognosis of KDSS, and reduce the sociomedical burden by accurately identifying high-risk KDSS groups and implementing more preventive interventions.

## 2 Materials and methods

### 2.1 Patient population

This retrospective study included a total of 74 children diagnosed with KDSS at Beijing Children’s Hospital, Capital Medical University, in 2021–2023. Two to three children with KD with stable hemodynamics at the same time (2 weeks before and after diagnosis) were randomly selected. A total of 156 children diagnosed with KD during the same period were also included. All children met the KD criteria proposed by the 2021 American College of Rheumatology/Vasculitis Foundation Guideline (1), the Japan Guideline written by Kobayashi et al. (7), and the KDSS criteria proposed by Kanegaye et al. (8) The exclusion criteria were as follows: (1) incomplete clinical data; and (2) the presence of serious underlying diseases (septic shock, anaphylactic shock, hypotension, coronary artery malformation, congenital heart disease.).

This study was approved by the Ethics Committee of Beijing Children’s Hospital affiliated with Capital Medical University (approval no. 2024-E-036-R).

### 2.2 Clinical data collection

The Jiahe platform system of Beijing Children’s Hospital was used to capture clinical data and establish KD database. No data screening or deletion was performed to ensure data integrity and objectivity. We collected data on sex, age, body mass index (BMI), fever duration, symptoms, and results of laboratory tests conducted prior to the onset of KDSS and treatment with intravenous immunoglobulin. All variables were collected within the first 12hrs of admission. Possible symptoms included maculopapular, diffuse erythroderma, or erythema multiforme–like rash; bilateral bulbar conjunctival injection without exudate; erythema and cracking of lips, strawberry tongue, and/or erythema of oral and pharyngeal mucosa; suppurative cervical lymph node enlargement; erythema and edema of the hands or feet (acute phase); and/or periungual desquamation (subacute phase). Laboratory tests included erythrocyte sedimentation rate (ESR), white blood cell count (WBC), absolute neutrophil count (ANC), platelet (Plt) count, hemoglobin (Hgb), C-reactive protein (CRP), high-sensitivity cardiac troponin I (hs_cTnI), brain natriuretic peptide (BNP), aspartate aminotransferase (AST), alanine aminotransferase (ALT), albumin (Alb), fibrinogen, total protein (TP), serum Na, serum K, serum Ca, serum P, Cr, total bile acid (TBA), glycocholic acid (CG), prothrombin time, international standard normalized ratio, partial thromboplastin time, thrombin time, d-dimer, antithrombin III activity. Echocardiographic results (left ventricular enlargement [LVE], decreased ejection fraction (DEF), presence or absence of pericardial effusion (PF), coronary artery dilatation (CAD).

### 2.3 Statistical analysis

A descriptive analysis was performed using SPSS version 26.0, while other statistical analyses were performed using R software (version 4.1.3). The data were randomly divided into a training set and a validation set at a 7:3 ratio. The training set was used for feature selection and model construction, while the validation set was used to evaluate the effectiveness of the training model.

#### 2.3.1 Univariate analysis

Normally distributed quantitative data are expressed as mean ± standard deviation, and intergroup comparisons were performed using the t-test. Non-normally distributed data are expressed as median and interquartile range, and groups were compared using the Mann–Whitney U Test test. Conversely, categorical data are expressed as frequency and percentage (%) and were compared using the χ^2^ test.

#### 2.3.2 Variable selection and prediction model establishment

A logistic regression model and a lasso regression model were used to screen predictors of KDSS. The logistic regression model used a stepwise method to screen variables (P < 0.05). Use of the lasso regression can reduce the collinearity between variables and ensure that the subsequently generated model is not overfitted. Considering the characteristic variables proposed by the above two methods, the five best variables were selected to establish a prediction model. A collinearity analysis was used to further determine whether there was an association between the included variables. A variance inflation factor (VIF) was used to quantify collinearity severity. Thereafter, variables without collinearity were included in the binary logistic regression analysis.

#### 2.3.3 Model performance evaluation

The accuracy of the model in the training set was assessed by receiver operating characteristic (ROC) curve. The discrimination of the model was determined by calculating the area under the ROC curve (AUC). The calibration curve was used to assess the goodness of fit between the predicted model and the observed data. The model’s calibration was assessed by comparison of the predicted and observed values and visualized using a calibration curve graph with 1000 bootstrap resampling procedures. The model was visualized by a nomogram.

#### 2.3.4 Model validation

The data from the test set were substituted into the model to predict the occurrence of KDSS. Calibration curves were plotted to verify the model’s accuracy and consistency, and its predictive value was assessed.

## 3 Results

A total of 74 children with confirmed KDSS admitted to the hospital in January 2021 to December 2023 were included in this study; another 156 children with KD admitted to the hospital during the same period were included. The training set included 52 children with KDSS and 110 children with KD, while the validation set included the remaining 22 children with KDSS and 46 children with KD. Variables whose data were incomplete were interpolated through the mice package.

### 3.1 Establishment of prediction model

#### 3.1.1 Univariate analysis

The t-, chi-squared, and Mann-Uhlenbeck tests used to conduct the univariate analysis of the clinical data revealed 24 statistically significant indicators (P < 0.05): BMI, rash, Hgb, Plt, ANC, CRP, BNP, hs_cTnI, K, Na, Ca, P, TP, Alb, Cr, AST, ALT, TBA, CG, FIB, LVE, DEF, PF, and CAD (**Table 1**).

**Table 1:** Characteristics of KDSS versus KD in the training set.

Factor	KDSS	KD	t/Z/*χ* ^2^ value	*P* value
Demographic parameters				
Number (%)	52 (32.1)	110 (67.9)	−	−
Male/female, n/n	33/19	66/44	0.178	0.673
Age (years), mean ± SD	4.87 ± 2.34	4.15 ± 2.10	-1.943	0.054
BMI (kg/m^2^), mean ± SD	16.38 ± 4.03	15.17 ± 2.08	-2.509	0.013
Clinical symptom parameters				
**Fever duration (days), mean ± SD**	6.27 ± 3.23	6.83 ± 2.28	1.265	0.208
Rash, n (%)	43 (82.7)	71 (64.5)	5.577	0.018
Eye, n (%)	51 (98.1)	108 (98.2)	0.002	0.963
Lip, n (%)	52 (100)	109 (99.1)	0.476	0.490
Lym, n (%)	50 (96.2)	103 (93.6)	0.426	0.514
Hand and foot, n (%)	39 (75.0)	95 (86.4)	3.189	0.074
Blood count parameters				
WBC (10^12^/L)	14.12 ± 6.88	13.55 ± 5.09	-0.588	0.557
Hgb (g/L)	109.23 ± 17.96	115.18 ± 10.35	2.668	0.008
Plt (10^9^/L)	221.19 ± 90.54	373.61 ± 123.31	7.952	<0.01
ANC (10^9^/L)	11.57 ± 6.06	8.40 ± 4.65	-3.659	<0.01
CRP (mg/L)	108 (71.25–158.50)	35.50 (18.75–55.75)	40.897	<0.01
ESR (mm/h)	74.50 (44.25–92.00)	77.00 (50.75–87.75)	0.015	0.904
BNP (pg/mL)	118.30 (80.70–292.75)	29.75 (16.48–48.78)	40.897	<0.01
HS-cTnI (ng/mL)	0.0110 (0.0043–0.0400)	0.0020 (0.0001–0.0040)	29.001	<0.01
Blood isochemical parameters				
K (mmol/L)	3.69 ± 0.50	4.18 ± 0.42	6.537	<0.01
Na (mmol/L)	130.80 ± 4.05	135.87 ± 2.50	0.776	<0.01
Ca (mmol/L)	2.02 ± 0.16	2.23 ± 0.15	8.123	<0.01
P (mmol/L)	0.97 ± 0.31	1.38 ± 0.19	10.554	<0.01
TP (g/L)	58.24 ± 8.22	65.10 ± 5.72	6.158	<0.01
Alb (g/L)	31.46 ± 4.50	37.47 ± 3.82	8.813	<0.01
Cr (µmol/L)	32.63 ± 15.76	25.49 ± 7.29	-3.948	<0.01
AST (U/L)	33.65 (24.13–45.90)	25.80 (21.68–31.95)	7.250	0.007
ALT (U/L)	24.85 (18.85–55.20)	14.45 (10.85–19.38)	45.315	<0.01
TBA (µmol/L)	14.89 (9.69–23.35)	5.20 (3.31–8.52)	36.705	<0.01
CG (mg/L)	5.30 (2.76–8.09)	2.08 (1.41–2.88)	22.204	<0.01
Coagulation parameters				
PT (s)	13.85 ± 2.06	13.41 ± 7.75	-0.403	0.688
INR	1.22 ± 0.18	1.18 ± 0.66	-0.409	0.683
Fib (g/L)	5.12 ± 1.81	4.63 ± 1.35	-2.126	0.035
APTT (s)	34.64 ± 3.85	35.34 ± 5.16	0.877	0.382
TT (s)	15.24 ± 3.21	15.24 ± 7.92	-0.007	0.995
Cardiac ultrasound parameters				
LVE, n (%)	23 (44.2)	3 (2.7)	45.141	<0.01
DEF, n (%)	19 (36.5)	0 (0.0)	45.533	<0.01
PF, n (%)	15(28.8)	8 (7.3)	13.490	<0.01
CAD, n (%)	16 (30.8)	16 (14.5)	5.863	0.020

Alb, albumin; ALT, alanine aminotransferase; ANC, absolute neutrophil count; APTT, partial thromboplastin time; AST, aspartate aminotransferase; BNP, brain natriuretic peptide; Ca, serum calcium; CAD, coronary artery dilatation; CG, glycocholic acid; Cr, creatinine; CRP, C-reactive protein; DEF, decreased ejection fraction; ESR, erythrocyte sedimentation rate; Eye, bilateral bulbar conjunctival injection without exudate; Fib, fibrinogen; Hand and foot, erythema and edema of the hands or feet (acute phase) and/or periungual desquamation (subacute phase); Hgb, hemoglobin; Hs-cTnI, high-sensitivity cardiac troponin I; INR, international normalized ratio; K, serum potassium; LVE, left ventricular enlargement; Lip, erythema and cracking of lips, strawberry tongue, and/or erythema of oral and pharyngeal mucosa; Lym, cervical lymphadenopathy (at least one lymph node >1.5 cm in diameter); Na, serum sodium; P, serum phosphorus; PF, pericardial fluid; Plt, platelet; PT, prothrombin time; Rash, maculopapular, diffuse erythroderma, or erythema multiforme–like rash; TBA, total bile acid; TP, total protein; TT, thrombin time; WBC, white blood cell.

#### 3.1.2 Multivariate analysis

Based on the univariate analysis results, 24 significant factors (P < 0.05) were identified on the multivariate analysis: BMI, rash, Hgb, Plt, ANC, CRP, BNP, hs_cTnI, K, Na, Ca, P, TP, Alb, Cr, AST, ALT, TBA, CG, FIB, LVE, EF, PF, and CAD.

**Figure 1:**
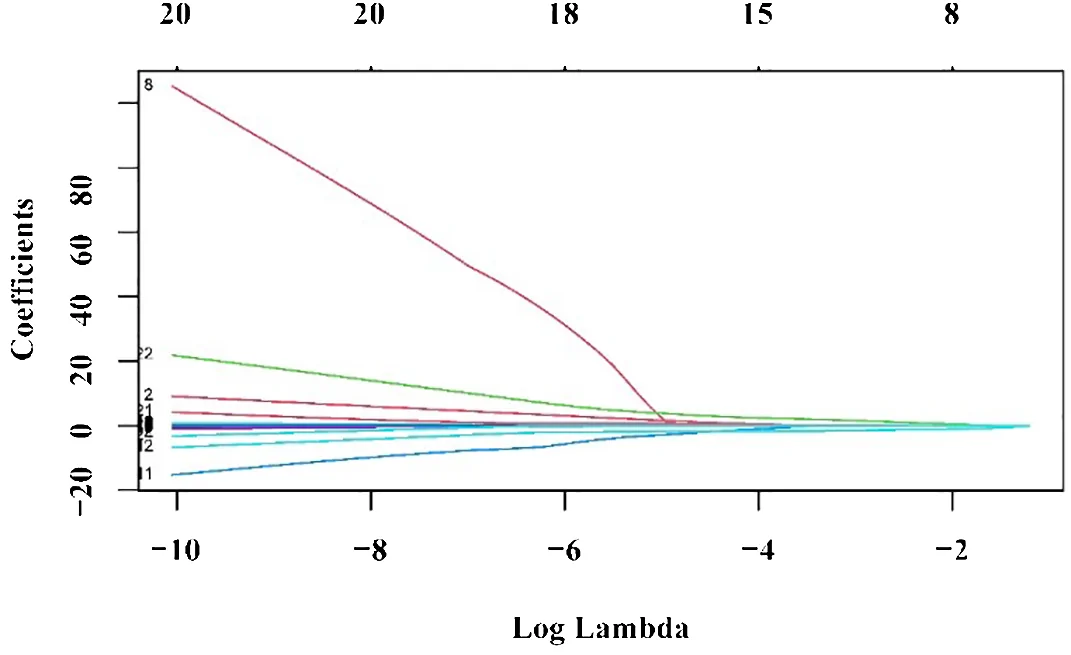
Lasso coefficient profiles of the 24 risk identified factors.

**Figure 2:**
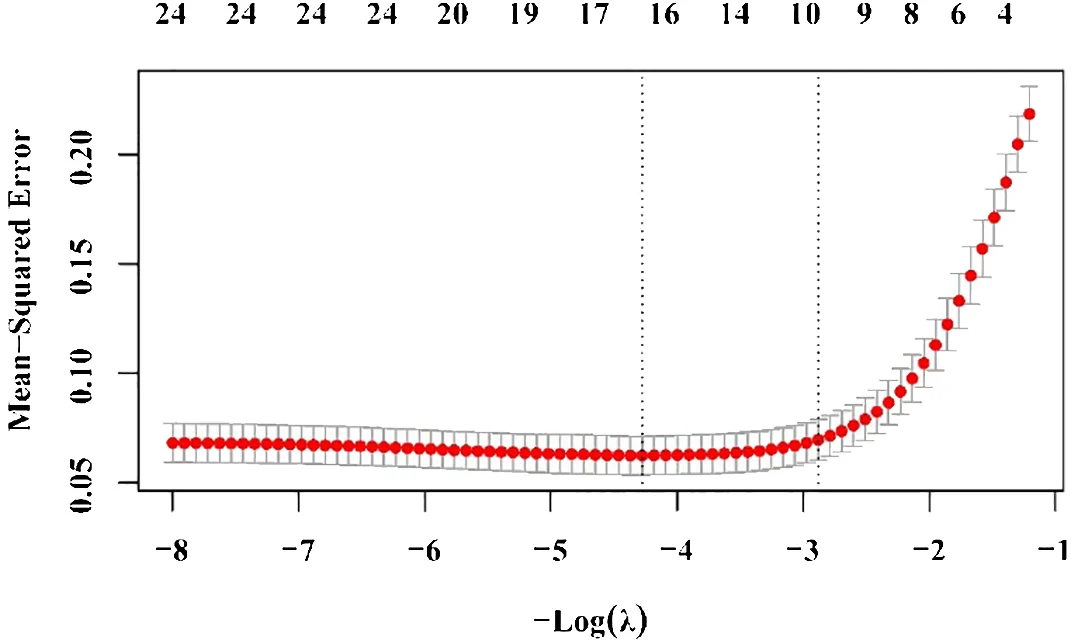
Ten risk factors selected by the lasso regression analysis. The two dotted vertical lines were drawn at the optimal scores by minimal criteria and 1-S.E. criteria (body mass index, rash, brain natriuretic protein, potassium, sodium, phosphorus, albumin, aspartate aminotransferase, alanine aminotransferase, and total bile acid). S.E., standard error.

Two regression methods were used to screen the variables. First, nine variables were screened in the logistic regression analysis, including rash, Plt, Na, Ca, P, Cr, AST, EF, and BNP. To prevent overfitting, we applied Lasso regression for variable selection once again. The following 10 indicators were screened by the lasso regression analysis: BMI, rash, BNP, K, Na, P, Alb, AST, ALT, and TBA (**Figures 1**, **2**). We screened five optimal variables from the two regression models, including rash, BNP, Na, P, and AST.

### 3.2 Collinearity analysis

Five variables, including rash, BNP, Na, P, and AST, were screened from the two regression models by the collinearity analysis. We use tolerances and VIF to quantify the collinearity severity. The tolerance of each variable was >0.2 and VIF was <5, indicating no significant collinearity between the two variables.

### 3.3 Prediction model formula

A prediction model was established based on logistic regression coefficient and constant terms to predict the risk of KDSS among children with KD. The logistic regression equation is as follows (**Table 2**):

**Table 2:** Coefficients of binary logistic regression for predicting KDSS among the training set.

Variable	*P* value	OR	95% CI for OR	
Rash	0.035	11.811	1.550–169.567	
BNP	0.000	1.026	1.015–1.042	
Na	0.001	0.628	0.461–0.799	
P	0.001	0.0038	0.0001–0.0739	
AST	0.068	1.045	1.000–1.101	
Constant	62.395	18.644	3.347	0.001

AST, aspartate aminotransferase; BNP, brain natriuretic peptide; CI, confidence interval; Na, serum sodium; OR, odds ratio; P, serum phosphorus; S.E., standard error.


Logit (P)=62.395+2.469×rash+0.026×BNP–0.466×Na–5.571×P+0.044×AST

### 3.4 Logistic regression models evaluated in the training set

**Figure 3:**
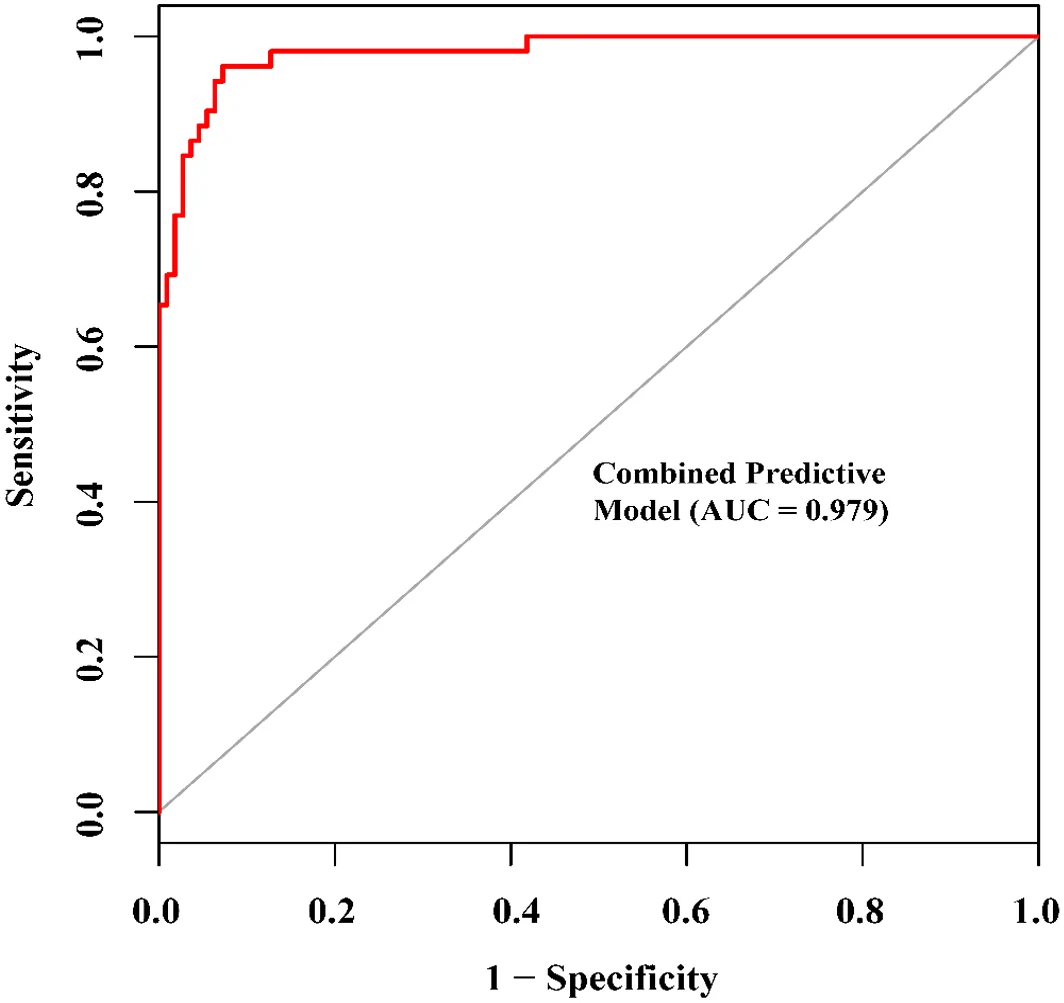
The ROC curve of the combined predictive model for predicting KDSS in training set.

**Figure 4:**
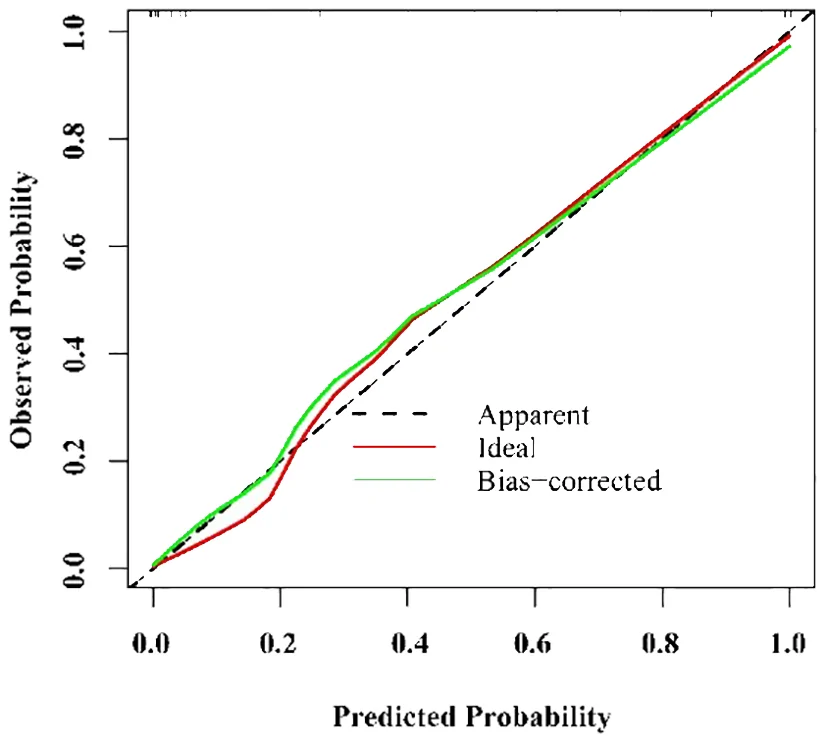
Calibration of nomogram for predicting Kawasaki disease shock syndrome in the training set.

A ROC curve analysis used to evaluate the discriminant performance of the logistic regression model revealed an AUC=0.979, sensitivity=96.2%, and specificity=97.2% (**Figure 3**). Consistency was checked using the calibration curve method. The calibration curve of the logistic regression model drawn in the training set showed that the calibration curve fit the standard curve well and the model calibration effect was good (**Figure 4**).

### 3.5 Establishment of nomogram model

**Figure 5:**
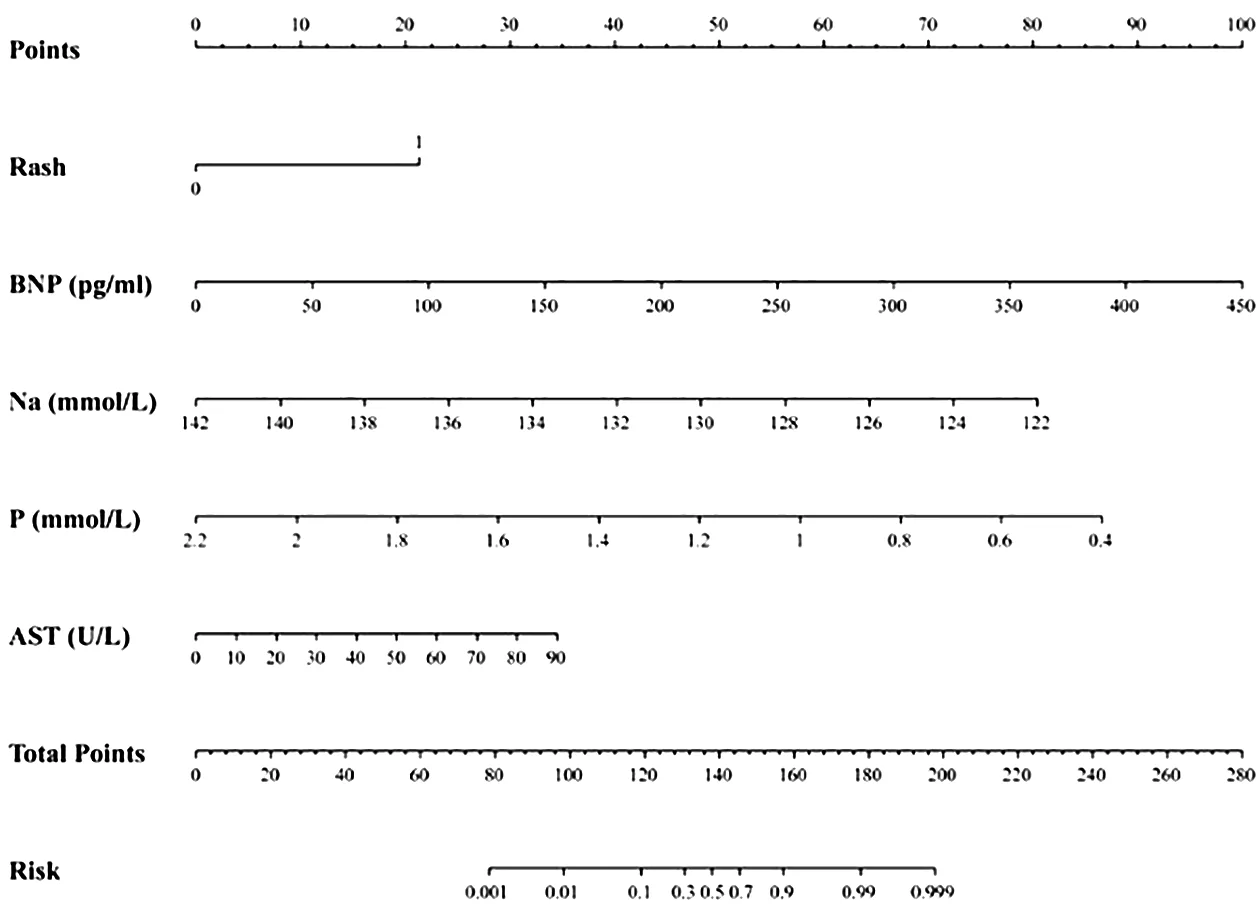
Nomogram for predicting Kawasaki disease shock syndrome among the training set.

According to the binary logistic regression analysis results, R software was used to construct a nomogram model (**Figure 5**) to visualize the model. To predict the incidence of KDSS, a line perpendicular to the axis of the corresponding index points was drawn on the column chart according to the values of rash, BNP, Na, P, and AST of each child with KD at admission, and the index points were summed. Next, the sum over the total number of points was determined, and a line plotted perpendicular to the risk axis to indicate the probability of KDSS occurring in children with KD.

### 3.6 Validation of logistic regression models in test sets

**Figure 6:**
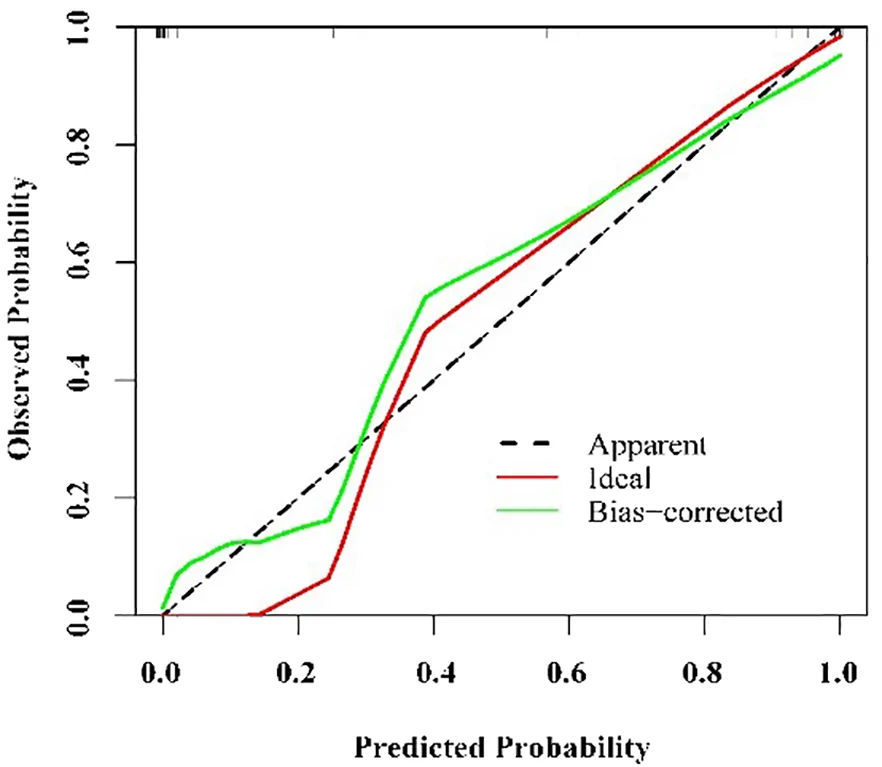
Calibration of nomogram for predicting Kawasaki disease shock syndrome among the testing set.

The calibration diagram of the test set showed that the predicted values of the logistic regression model were in good agreement with the actual values (**Figure 6**). The correct prediction rate of the model is 1 – (4/68) = 94.12% (**Table 3**).

**Table 3:** Predictive values of nomogram model for internal validation set.

Nomogram model-predicted efficacy outcome	Clinical standard-based follow-up outcome		Total
	KDSS	KD	
KDSS	19	1	20
KD	3	45	48
Total	22	46	68

KD, Kawasaki disease; KDSS, Kawasaki disease shock syndrome.

## 4 Discussion

KDSS is a severe subtype of KD that features acute onset and severe illness. Because KD shares features with other febrile illnesses in childhood, many common symptoms have been identified (9) that prevents its easy recognition. The early manifestation of KDSS is incomplete, and it is easily misidentified. Therefore, the best time for diagnosis and treatment is commonly missed, which can lead to death in severe cases. The incidence of KDSS is low, and the study with the largest sample size in China was from Taiwan, where the incidence of KDSS in KD was 1.45% (10), much lower than the 2.8–5.3% reported in the United States (3), adding to difficulty understanding the disease. KD can involve the coronary system, and studies have shown that the risk of coronary artery injury is higher in KDSS than in KD (11). The lack of the early recognition of KDSS and delayed treatment may lead to permanent coronary structural damage (12), thereby increasing the risk of long-term complications. Therefore, the early diagnosis and treatment of KDSS is particularly important.

Rather than screening for the most relevant risk factors, this study aimed to screen for the most parsimonious risk factors from among the many independent risk factors of KDSS and construct the best logistic regression model to predict early KDSS.

The incidence of KDSS is low; in fact, only 74 children with KDSS were enrolled in the largest pediatric hospital in China over the past 3 years. Considering the limited sample size and as many as 24 clinical/laboratory variables as being statistically significant, traditional methods may be unsatisfactory. We used a logistic regression stepwise method and a lasso regression analysis to screen variables together. Next we obtained the best data introduce into the model. Finally, we screened out five variables, including rash, BNP, Na, P, and AST. We found that rash, BNP, hyponatremia, hypophosphatemia, and AST were independent risk factors for KDSS. These findings both support the existing hypothesis on the pathogenesis of KDSS and provide new insight into its diagnosis and management.

Rash was identified as an independent risk factor for KDSS, a finding that is consistent with the previous literature (2). A persistent extensive rash may reflect a more severe systemic, aggravating increased microvascular permeability and capillary leakage.

BNP concentration is a quantitative plasma biomarker for the presence and severity of hemodynamic cardiac stress and heart failure (HF). End-diastolic wall stress, intracardiac filling pressure, and cardiac volume appear to be its main triggers. BNP has high prognostic accuracy for a patient’s risk of death and HF hospitalization (13). KD is a systemic vasculitis disorder. In animal models of KD, Immunoglobulin A and C3 immune complexes are deposited within the cardiovascular tissue (14). KDSS is a more severe vasculitis than KD. In contrast, myocardial injury in KDSS is more severe than that in KD. As described in this article, LVE in children with KDSS was 44.2%, while LVE in children with KD was 2.7%, with a significant statistical difference (P < 0.01). Ejection fraction in children with KDSS was significantly higher than that in children with KD, confirming that the former have more severe cardiac dysfunction. BNP is synthesized and released by ventricular myocardial cells in response to stress on myocardial walls caused by volume or pressure overload and ischemia. In addition to hemodynamic stress, inflammation in myocardial tissue can induce BNP production (15). Therefore, BNP may be a sensitive indicator of KDSS.

Hyponatremia and hypophosphatemia are two electrolyte disorders associated with KDSS. In patients with KDSS, persistent hypotension and inadequate effective circulatory volume may lead to an increased renal excretion of Na and P, leading to hyponatremia and hypophosphatemia. Hypophosphatemia can be considered the superposition of myocardial depression, peripheral vascular dilation insufficiency, and acidosis in shock. Hypophosphatemia occurs in many critically ill patients and usually indicates severe disease (16, 17). One study showed (18) that, among patients with sepsis but without CKD, the risk of death and shock in patients with hypophosphatemia was significantly higher than that in patients without hypophosphatemia. The conditions of patients with KD and hyponatremia are generally more serious than those of patients without hyponatremia. In particular, in terms of the incidence of coronary artery lesions, the odds ratio of patients with KD with versus without hyponatremia was as high as 4.78 (19). These electrolyte disorders may further affect cardiovascular function, aggravate shock symptoms, and even be life-threatening in severe cases. These findings are consistent with those of this study. Sodium is a key electrolyte in maintaining intra- and extracellular water balance and neuromuscular function. Hyponatremia may lead to neurological symptoms such as headache, nausea, convulsions, and even coma. Phosphorus is an essential electrolyte for maintaining intra- and extracellular energy metabolism and bone health. Hypophosphatemia may cause symptoms such as muscle weakness and arrhythmia.

AST, an enzyme found in the liver and other tissues, is not a specific indicator of KDSS. AST is mainly found in the mitochondria of cells in the liver, kidneys, brain, lungs, and skeletal muscle (20). KDSS, a serious complication of KD characterized by persistent hypotension and inadequate effective circulation, leads to multiorgan dysfunction of the digestive, respiratory, and nervous systems (21) and further promotes an elevated AST. Some reports suggest that elevated AST levels may be associated with hepatocyte damage caused by inflammation (11). In recent years, the ratio of AST and ALT levels, which usually indicate chronic liver disease severity, can predict prognosis (22, 23). Adult patients with higher baseline AST/ALT levels are more likely to develop fatal cardiovascular disease (24). Other studies demonstrated that AST/ALT is a risk factor for coronary artery injury at admission (25). This supports the possible association between AST and a severe inflammatory response.

These findings encourage the further exploration of potential new mechanisms of KDSS pathogenesis. It is worth noting that our model is the most rigorously validated for KDSS predictions. The model performed well in the training set (AUC=0.979) and achieved good differential diagnostic performance in the validation set. The calibration curve analysis further confirmed its reliability for practical application.

Our study is the first to integrate multiple clinical indicators into a prediction model, thus providing a powerful tool for the early identification of KDSS. Compared with a single biomarker, the model comprehensively considers multiple pathophysiological processes, giving it higher discrimination ability. The risk of developing KDSS can be determined according to the results of routine examinations on admission, which is conducive to the timely implementation of individualized treatment and thereby reduces the risk of complications.

Certainly, our study has some shortcomings: 1) as a single-center study, the universality of the model requires verification in large-scale multi-center studies; 2) only clinical routine indicators are used at present, and model performance may be further improved in the future by the integration of multimodal data such as imaging findings; and 3) we focused on model construction, but the explanation of the underlying etiology and pathogenesis of KDSS remains insufficient, and further mechanism research of a larger sample is needed.

In summary, our multi-biomarker prediction model based on large-scale data is a valuable tool for the early identification of KDSS that lays a foundation for elucidating the heterogeneity of the disease and provides a new idea for clinical transformation. Compared with existing studies, our work made breakthroughs in sample size, validation rigor, application value evaluation, and other aspects. We have reason to believe that, through its continuous efforts to improve the model and explore the pathogenesis of KDSS in depth, it will make a major contribution to the accurate diagnosis and treatment of KDSS in children.

## Data availability statement

The original contributions presented in the study are included in the article/supplementary material. Further inquiries can be directed to the corresponding author.

## Ethics statement

The studies involving humans were approved by Beijing Children’s Hospital, Capital Medical University, National Center for Children’s Health China. The studies were conducted in accordance with the local legislation and institutional requirements. The human samples used in this study were acquired from primarily isolated as part of your previous study for which ethical approval was obtained. Written informed consent for participation was not required from the participants or the participants’ legal guardians/next of kin in accordance with the national legislation and institutional requirements.

## Author contributions

ZZ: Conceptualization, Data curation, Formal analysis, Funding acquisition, Investigation, Methodology, Project administration, Resources, Software, Supervision, Validation, Visualization, Writing – original draft, Writing – review & editing. YY: Conceptualization, Writing – original draft. LG: Project administration, Writing – review & editing. QL: Supervision, Writing – review & editing. YW: Formal analysis, Writing – review & editing. SZ: Data curation, Methodology, Supervision, Writing – review & editing.
