# Correction: Predicting Kawasaki disease shock syndrome in children

**DOI:** 10.3389/fimmu.2026.1925394

**Published:** 2026-09-03

**Authors:** Zhihui Zhao, Yue Yuan, Lu Gao, Qirui Li, Ying Wang, Shunying Zhao

**Affiliations:** Beijing Children’s Hospital, Capital Medical University, National Center for Children’s Health, Beijing, China

**Keywords:** Kawasaki disease shock syndrome, Kawasaki disease, logistic regression model, nomogram model, early identification

There was a mistake in **Figure 3** as published. An incorrect image file was inadvertently used for **Figure 3**, along with an incorrect caption.

**Figure 3:**
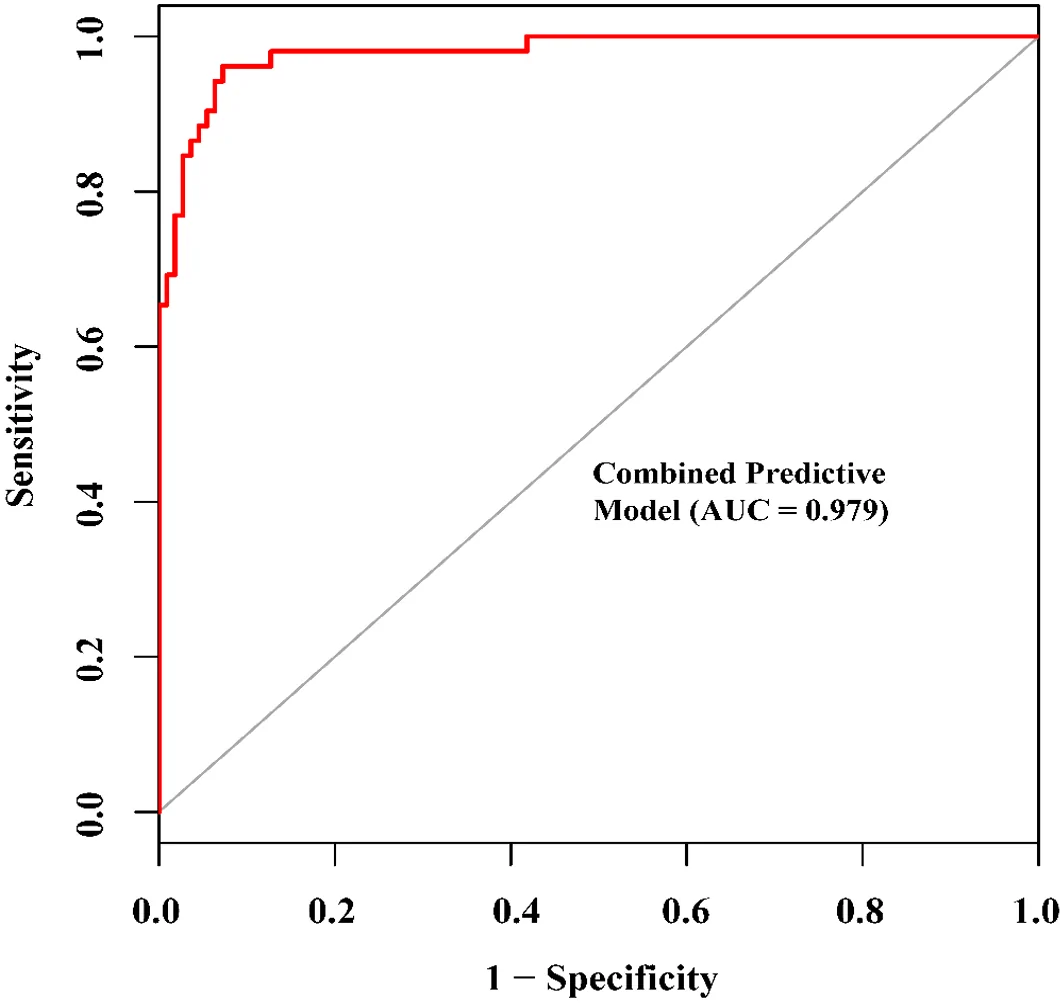
The ROC curve of the combined predictive model for predicting KDSS in training set.

The corrected **Figure 3** and its caption appears below.

The authors apologize for this error and state that this does not change the scientific conclusions of the article in any way. The original version of this article has been updated.

